# Integrative Analysis of Proteomics and DNA Methylation in Orbital Fibroblasts From Graves’ Ophthalmopathy

**DOI:** 10.3389/fendo.2020.619989

**Published:** 2021-02-15

**Authors:** Sita Virakul, Poorichaya Somparn, Trairak Pisitkun, Peter J. van der Spek, Virgil A. S. H. Dalm, Dion Paridaens, P. Martin van Hagen, Nattiya Hirankarn, Tanapat Palaga, Willem A. Dik

**Affiliations:** ^1^ Department of Microbiology, Faculty of Science, Chulalongkorn University, Bangkok, Thailand; ^2^ Center of Excellence in Systems Biology, Research affairs, Faculty of Medicine, Chulalongkorn University, Bangkok, Thailand; ^3^ Translational Research in Inflammation and Immunology Research Unit (TRIRU), Department of Microbiology, Faculty of Medicine, Chulalongkorn University, Bangkok, Thailand; ^4^ Department of Bioinformatics, Erasmus University Medical Center, Rotterdam, Netherlands; ^5^ Department of Immunology, Laboratory Medical Immunology, Erasmus University Medical Center, Rotterdam, Netherlands; ^6^ Department of Internal Medicine, Division of Clinical Immunology, Erasmus University Medical Center, Rotterdam, Netherlands; ^7^ Rotterdam Eye Hospital, Rotterdam, Netherlands; ^8^ Department of Ophthalmology, Erasmus Medical Center, Rotterdam, Netherlands; ^9^ Center of Excellence in Immunology and Immune Mediated Diseases, Department of Microbiology, Faculty of Medicine, Chulalongkorn University, Bangkok, Thailand

**Keywords:** graves’ ophthalmopathy, orbital fibroblast, proteomics, DNA methylation, epigenetics

## Abstract

**Background:**

Graves’ ophthalmopathy (GO) is a frequent extrathyroidal complication of Graves’ hyperthyroidism. Orbital fibroblasts contribute to both orbital tissue inflammation and remodeling in GO, and as such are crucial cellular elements in active GO and inactive GO. However, so far it is largely unknown whether GO disease progression is associated with functional reprogramming of the orbital fibroblast effector function. Therefore, the aim of this study was to compare both the proteome and global DNA methylation patterns between orbital fibroblasts isolated from active GO, inactive GO and healthy controls.

**Methods:**

Orbital fibroblasts from inactive GO (n=5), active GO (n=4) and controls (n=5) were cultured and total protein and DNA was isolated. Labelled and fractionated proteins were analyzed with a liquid chromatography tandem-mass spectrometer (LC-MS/MS). Data are available *via* ProteomeXchange with identifier PXD022257. Furthermore, bisulphite-treated DNA was analyzed for methylation pattern with the Illumina Infinium Human Methylation 450K beadchip. In addition, RNA was isolated from the orbital fibroblasts for real-time quantitative (RQ)-PCR. Network and pathway analyses were performed.

**Results:**

Orbital fibroblasts from active GO displayed overexpression of proteins that are typically involved in inflammation, cellular proliferation, hyaluronan synthesis and adipogenesis, while various proteins associated with extracellular matrix (ECM) biology and fibrotic disease, were typically overexpressed in orbital fibroblasts from inactive GO. Moreover, orbital fibroblasts from active GO displayed hypermethylation of genes that linked to inflammation and hypomethylated genes that linked to adipogenesis and autoimmunity. Further analysis revealed networks that contained molecules to which both hypermethylated and hypomethylated genes were linked, including NF-κB, ERK1/2, Alp, RNA polymerase II, Akt and IFNα. In addition, NF-κB, Akt and IFNα were also identified in networks that were derived from the differentially expressed proteins. Generally, poor correlation between protein expression, DNA methylation and mRNA expression was observed.

**Conclusions:**

Both the proteomics and DNA methylation data support that orbital fibroblasts from active GO are involved in inflammation, adipogenesis, and glycosaminoglycan production, while orbital fibroblasts from inactive disease are more skewed towards an active role in extracellular matrix remodeling. This switch in orbital fibroblast effector function may have therapeutic implications and further studies into the underlying mechanism are thus warranted.

## Introduction

Fibroblasts are crucial for maintaining tissue homeostasis, and are major producers of important cellular mediators for inflammatory and tissue remodeling processes during normal healing responses, but also under pathological conditions, including chronic inflammatory and fibrotic diseases (1). Chronic tissue inflammation and fibrosis are characterized by excessive fibroblast accumulation in affected tissues (1). Fibroblast accumulation occurs through different mechanisms, including enhanced proliferation by tissue resident CD34^-^ fibroblasts, recruitment of fibrocytes (a population of circulating cells with fibroblast-like properties that express CD34^+^ and extracellular matrix (ECM) molecules) and diminished apoptosis/prolonged survival (2–4). These fibroblasts can alter their phenotype and effector functions, as evidenced by their differentiation into myofibroblasts (5, 6). Myofibroblasts are considered to represent the end-effector cells in fibrotic reactions. Myofibroblasts express α-smooth muscle actin that facilitates cellular contraction and tissue distortion and actively produce high amounts of ECM molecules, such as collagen and hyaluronan (1).

Graves’ ophthalmopathy (GO) is a disease of the orbital soft-tissues surrounding the eyes, and affects up to 50% of patients with Graves’ disease (GD) (7). In general, GO patients first suffer from an initial phase of progressive disease (the ‘active’ phase) that is characterized by active inflammation (8). This active phase may last for months after which the activity subsides and progresses to a phase of slow spontaneous recovery. This ‘chronic’ phase may take months to years and is associated with pathological tissue alterations, including adipose tissue expansion, excessive hyaluronan accumulation and fibrosis (8). The orbital tissue alterations are largely responsible for several morbidities such as proptosis, chronic eye movement dysfunction, and eventually determines the ‘severity’ of GO (7). Previously, protein expression profiles of orbital tissues from GO with different disease activity and smoking status revealed differences in several proteins involved in inflammation and adipogenesis (9, 10). However, those proteins could not be specifically linked to their pathogenic cellular source.

Orbital fibroblasts represent the main effector cells in GO as they are crucially involved in regulation of both the local inflammatory and tissue remodeling responses. The effector functions of the orbital fibroblasts are in turn affected by several mediators, including the thyrotropin receptor (TSHR) stimulatory autoantibodies, cytokines, growth factors and physical cellular interactions (8). Importantly, orbital fibroblasts from GO patients may react in a different way to such stimuli than orbital fibroblasts isolated from healthy orbital tissue. For instance a stronger upregulation of CD40, Thy1, IGF1R and BAFF by GO orbital fibroblasts was reported (11–14). Moreover, orbital fibroblasts isolated from active GO display enhanced secretion of certain pro-inflammatory mediators in comparison to orbital fibroblasts from control tissue, even in the absence of further *in vitro* stimulation (15, 16). Furthermore, differentiation of orbital fibroblasts into adipocytes and pro-fibrotic myofibroblasts appears to be more restricted to the late inactive stage of disease and is associated with increased TSHR expression by these cells (17, 18).

Gradually, epigenetic regulation (e.g. histone modification, DNA methylation and non-coding RNA) has become the subject of studies in primary fibroblasts isolated from different fibroproliferative diseases, including idiopathic pulmonary fibrosis (IPF) and systemic sclerosis (SSc) (4, 19–22). Fibroblasts from IPF and SSc have altered DNA methylation profiles, which contribute to the regulation of gene transcription in these cells (19, 20). In GO, differential gene expression has been demonstrated in orbital tissue, suggesting a role for epigenetics in the pathogenesis of GO (23). However, data on DNA methylation in orbital fibroblasts from GO is lacking to date. Insight in this could help to develop novel therapeutic options that target aberrant epigenetic modifications in GO. Here we conducted a study to compare the proteome of orbital fibroblasts isolated from active GO, inactive GO and control orbital tissue and integrated these data with global DNA methylation analysis performed on the same orbital fibroblasts.

## Material and Methods

### Orbital Fibroblast Isolation From Orbital Tissue

Orbital fibroblasts were cultured from four patients with GO in an active stage (clinical activity score (CAS) ≥ 3/7) and five patients with GO in an inactive stage of disease (CAS < 3/7) (24), who underwent orbital decompression surgery. In addition, orbital fibroblasts were cultured from five controls without thyroid or inflammatory disease that underwent orbital surgery for other reasons, as described previously (25). GO patients were euthyroid and had not received immunosuppressive treatment for at least three months prior to orbital decompression surgery. Further patient characteristics are given in **Table 1**. All orbital tissues were obtained at the Rotterdam Eye Hospital (Rotterdam, the Netherlands), after informed consent and in accordance with the principles of the Declaration of Helsinki. Approval was given by the local medical ethics committee (protocol ID-2007-01). Orbital fibroblasts were cultured in Dulbecco’s modified Eagle’s medium (DMEM) supplemented with 10% fetal calf serum (FCS) and antibiotics (penicillin and streptomycin; Cambrex BioWhittaker, Verviers, Belgium) (25). Orbital fibroblasts were serially passaged with gentle treatment of trypsin/EDTA and used for experiments between the 2^nd^ and 6^th^ passage.

**Table 1:** Patients and controls data.

Participants	Cell ID	Age	Gender
*Healthy control*	**COF-3**	48	Female
	**COF-4**	82	Female
	**COF-7**	80	Female
	**COF-9**	59	Female
	**COF-10**	37	Female
*Inactive GO*	**GO-11**	43	Female
	**GO-27**	69	Female
	**GO-31**	46	Female
	**GO-56**	51	Female
	**GO-89**	32	Female
*Active GO*	**GO-19**	31	Female
	**GO-37**	62	Female
	**GO-88**	77	Male
	**GO-90**	84	Female

### Proteome Analysis

#### Protein Extraction, Peptide Preparation and Labeling

Orbital fibroblasts from four patients with GO at an active stage and five GO patients at an inactive stage of disease, and five controls were included in this experiment. Orbital fibroblasts (5 x 10^6^ cells) were obtained and pelleted after which Halt™ Protease Inhibitor Cocktail and Phosphatase Inhibitor Cocktail (Thermo Fisher Scientific, Rockford, IL) was added before digestion and homogenization in 5% sodium deoxycholate with a sonicator. Protein concentration was determined with Pierce™ BCA Protein Assay Kit (Thermo Fisher Scientific, Rockford, IL). For peptide preparation, 100 µg of protein was digested with 2 µg trypsin and labelled with 10-plex tandem mass tag (TMT) reagent according to the manufacturer’s protocol (Thermo Fisher Scientific, Rockford, IL). Afterwards, the peptide mixture was passed through the Pierce High pH Reversed-Phase Peptide Fractionation Kit (Thermo Fisher Scientific, Rockford, IL).

### Peptide Analysis

The labelled fractionated peptides were analyzed with a liquid chromatography tandem-mass spectrometer (LC-MS/MS) (Q Exactive Plus mass spectrometer; Thermo Scientific, San Jose, CA). The LC-MS/MS methods included a full MS scan at a resolution of 70,000 followed by 10 data-dependent MS2 scans at a resolution of 37,500. The normalized collision energy of higher-energy collisional dissociation (HCD) fragmentation was set at 28%. MS scan range of 400 to 1,600 m/z was selected and precursor ions with unassigned charge states, a charge state of +1 and a charge state of greater than +8 were excluded. Proteome discoverer 2.1 software (Thermo Scientific, San Jose, CA) was used to analyze the MS raw data files with database containing forward and reverse peptide sequences from the human Uniprot Database. The search parameters were set for the fix modifications of carbamidomethylation of cysteine (+57.02146 Da) and TMT modifications (+229.2634 Da) at N-terminal and lysine, while oxidation of methionine (+15.99491 Da) was set for the variable modification. A maximum of four modifications and two missed cleavages per peptide were allowed. Parent and fragment monoisotopic mass errors were set at 10 ppm and 0.2 Da, respectively. A target–decoy approach was used to limit the false discovery rate (FDR) of the identified peptides to less than 1%. Differential protein expression was compared between orbital fibroblasts isolated from active and inactive GO by Student’s t-test. The mass spectrometry proteomics data have been deposited to the ProteomeXchange Consortium *via* the PRIDE [1] partner repository with the dataset identifier PXD022257. Network, pathway and functional analysis were further performed on these differential expressed proteins using Ingenuity (Qiagen) filtering only for those networks and pathways that are based on experimentally obtained information.

### DNA Methylation Analysis

Total DNA was extracted from orbital fibroblasts of active GO (n = 4), inactive GO (n = 4) and controls (n = 4). Global DNA methylation was measured using the Illumina Infinium Human Methylation 450K beadchip on bisulphite-treated DNA. DNA methylation analysis was performed by GenomeStudio methylation analysis package. Methylation level ranges from 0 (unmethylated) to 1 (fully methylated). Differential DNA methylation levels/patterns were analyzed by two approaches; 1) genes with differential DNA methylation using FDR < 0.05, and 2) differences in DNA methylation level of at least 2-fold between active and inactive GO. This was followed by network, pathway and functional analysis using Ingenuity (Qiagen) filtering only for those networks and pathways that are based on experimentally obtained information.

### mRNA Expression by Orbital Fibroblasts

Messenger RNA was isolated (GenElute Mammalian Total RNA Miniprep Kit; Sigma-Aldrich, St. Louis, MO, USA) from orbital fibroblasts from the same culture experiment as used for the proteome and DNA methylation studies. The isolated mRNA was converted into cDNA and gene expression was determined by real-time quantitative (RQ)-PCR (QuantStudio 5 Real-Time PCR System; Applied Biosystems, Waltham, MA) and normalized to the control gene *ABL*, as described previously (25). Primer-probe combinations (*ABL*) and TaqMan gene expression assays (Life technologies, Foster, CA) used are listed in **Supplementary Table 1**. Data from mRNA expression was analyzed using ANOVA and subsequently analyzed with the Mann Whitney U test. A P-value < 0.05 was considered statistically significant.

## Results

### Proteome Analysis in Orbital Fibroblasts

**Figure 1:**
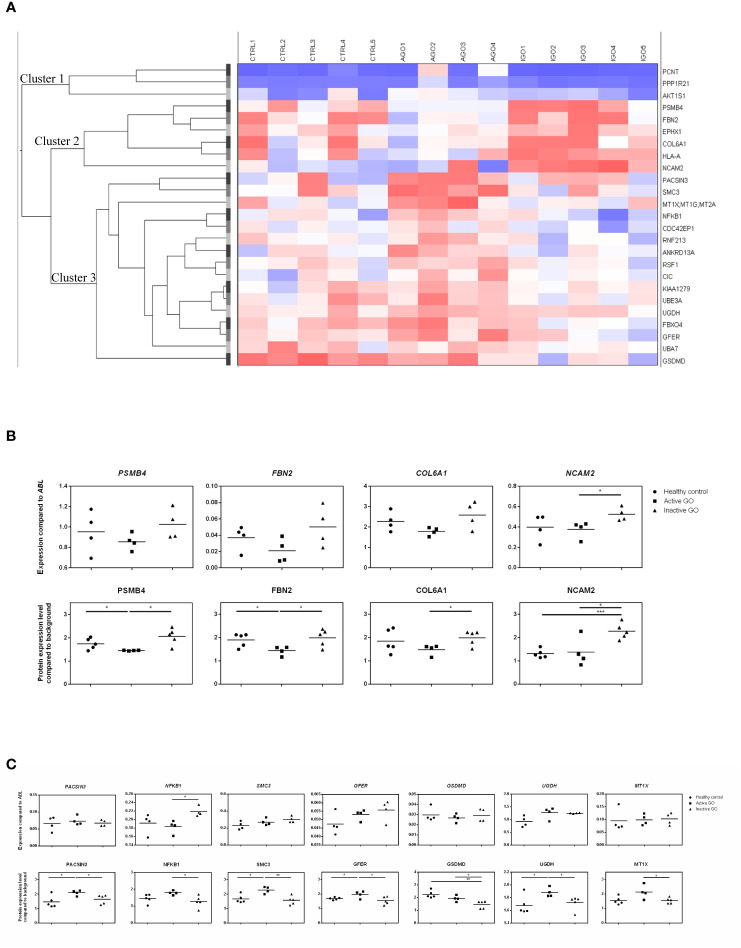
Proteomic profiles of orbital fibroblasts from inactive GO, active GO and healthy controls. **(A)** Cluster analysis of the differential protein expression between orbital fibroblasts from patients with inactive GO (n = 5), active GO (n = 4) and healthy controls (n = 5). Differentially expressed proteins grouped in three main clusters. Proteins within cluster 1 proteins were expressed at slightly higher level in orbital fibroblasts from active GO compared with orbital fibroblasts from inactive GO and controls. Proteins within cluster 2 displayed higher expression in orbital fibroblasts from inactive GO compared with orbital fibroblasts from active GO and controls. Proteins within cluster 3 were expressed at a higher level in orbital fibroblasts from active GO compared to inactive GO and controls. **(B)** Gene and protein expression level from proteins within cluster 2. Protein expression levels were compared to high abundant protein expression level. Four proteins (PSMB4, FBN2, COL6A1 and NCAM2) up-regulated in inactive GO orbital fibroblasts compared to active GO were related to extracellular matrix biology and further determined by RQ-PCR. * and *** indicate p-value < 0.05 and < 0.001, respectively. Individual symbols represent orbital fibroblast cultures from individual patients. Horizontal bar depicts the median. **(C)** Gene and protein expression level from proteins within cluster 3. Protein expression levels were compared to high abundant protein expression level. Seven proteins (PACSIN3, NFKB1, SMC3, GFER, GSDMD, UGDH and MT1X) down-regulated in inactive GO orbital fibroblasts compared to active GO related to inflammation, hyaluronan and adipogenesis were determined by RQ-PCR. * and ** indicate p-value < 0.05 and < 0.01, respectively. Individual symbols represent orbital fibroblast cultures from individual patients. Horizontal bar depicts the median.

Twenty-five proteins differed significantly in their expression level between orbital fibroblasts from patients with active GO versus inactive GO (**Supplementary Table 2**). Cluster analysis revealed that the differentially expressed proteins clustered into three main groups (**Figure 1A**, indicated as cluster 1, cluster 2 and cluster 3). Proteins (n=6) in cluster 2 displayed higher expression in orbital fibroblasts from inactive GO compared to orbital fibroblasts from active GO and controls (**Figure 1B**). The proteins (n=16) in cluster 3 were higher expressed in orbital fibroblasts from active GO compared to inactive GO and controls (**Figure 1C**). The proteins (n=3) in cluster 1 were expressed at slightly higher level in orbital fibroblasts from active GO than orbital fibroblasts from inactive GO and controls.

### Validation of Differential Protein Expression by RQ-PCR

For eleven of the proteins found to be differentially expressed (4 up-regulated and 7 down-regulated in inactive GO orbital fibroblasts compared to active GO) the expression levels of the corresponding mRNA molecules were determined by RQ-PCR (**Figures 1B, C** **)**. From the 4 proteins (PSMB4, FBN2, COL6A1 and NCAM2) upregulated, only *NCAM2* mRNA was significantly higher expressed (P<0.05) in orbital fibroblasts from inactive GO compared to active GO (**Figure 1B**), while the mRNA levels of *PSMB4*, *FBN2* and *COL6A1* only showed a trend to higher expression in inactive GO orbital fibroblasts compared with active GO orbital fibroblasts (**Figure 1B**). Of the 7 proteins (PACSIN3, NFKB1, SMC3, GFER, GSDMD, UGDH and MT1X) that were expressed at significantly lower levels in orbital fibroblasts from inactive GO, none of the corresponding mRNAs was significantly decreased in inactive GO (**Figure 1C**). Unexpectedly, *NFKB1* mRNA expression was significantly up-regulated in inactive GO orbital fibroblasts compared to active GO (**Figure 1C**).

### Orbital Fibroblast Expressed Proteins in Active GO Relate to Inflammation, Hyaluronan and Adipogenesis

Proteins overexpressed by orbital fibroblasts from active GO related to inflammation, hyaluronan synthesis and adipogenesis. One of the inflammation related proteins highly expressed in active GO orbital fibroblasts was NFKB1 (**Figure 1C**). The NFKB1 105 kD protein can be processed by the 26S proteasome into the DNA binding p50 subunit of the transcription factor NF-κB (26). NF-κB stimulates expression of genes involved in inflammation in many different cell types, including orbital fibroblasts (27, 28). The NFKB1 subunit exerts a protective role by dampening inflammation (29). Therefore, upregulation of NFKB1 may represent a counter regulatory mechanism to control the inflammatory response in orbital fibroblasts from active GO (8, 11).

ECM and fibrosis related proteins that were overexpressed in orbital fibroblasts from active GO included structural maintenance of chromosomes 3 (SMC3), growth factor, augmenter of liver regeneration (GFER) and uridine diphosphate-glucose dehydrogenase (UGDH) (**Figure 1C**). UGDH is involved in the biosynthesis of glycosaminoglycans (GAG), such as hyaluronan, chondroitin sulfate and heparan sulfate and was previously found elevated in urine from patients with active GO (30). Moreover, UGDH was found to be expressed at higher levels in orbital fibroblasts than in fibroblasts from other anatomical regions (31). This indicates that orbital fibroblasts in active GO acquire a phenotype very well equipped for GAG synthesis (32). Orbital fibroblasts from active GO also produced more protein kinase c and casein kinase substrate in neurons (PACSIN) 3, a protein involved in clathrin-mediated endocytosis that regulates glucose uptake by adipocytes (33, 34). The up-regulation of PACSIN3 in the active orbital fibroblasts may therefore relate to formation of adipocytes that finally accumulate in the inactive phase of GO (35).

### Orbital Fibroblast Expressed Proteins in Inactive GO Relate to Extracellular Matrix Biology

Orbital fibroblasts from inactive GO overexpressed several proteins linked to ECM biology and fibrotic diseases. This included fibrillin-2 (FBN2), collagen type VI alpha 1 chain (COL6A1) and the neural adhesion molecule 2 (NCAM2) (**Figure 1B**) (36–39). FBN2 is involved in elastic fiber formation and is abundantly present in embryonic tissues, but is also elevated along with elastin in fibrotic tissue, for instance in systemic sclerosis (36, 40). Yet, data on elastin accumulation in orbital tissue from GO patients is lacking so far. The non-fibrillar collagen type VI is involved in structural organization of the ECM, for instance by interacting with a multitude of other key ECM components, including the fibrillary type-1 and type 3 collagens and hyaluronan (39, 41, 42). Increased COL6A1 tissue levels are present in lung fibrosis, liver fibrosis and keloid scarring (37–39). Furthermore, collagen type VI can bind a variety of different growth factors implicated in fibrosis, and as such serves as a reservoir that can regulate growth factor activity in the vicinity of fibroblasts and contribute to fibrosis (39, 42, 43). Although data on collagen type VI in GO orbital tissue is lacking so far, several growth factors that bind collagen type VI, including PDGF-AB, PDGF-BB, and HGF have been linked to GO pathogenesis (44–46). Neural adhesion molecule 2 (NCAM2) was also higher expressed by orbital fibroblasts from inactive GO. NCAM2 can activate fibroblast growth factor receptor (FGFR), and recent studies demonstrated that FGFR activation in orbital fibroblasts stimulates hyaluronan synthesis and adipogenesis (47–49). Therefore, it can be postulated that in late inactive GO, orbital fibroblast-derived NCAM2 might be involved in adipogenesis and hyaluronan synthesis through FGFR activation. Clearly further detailed study on the role of the molecules we here found to be expressed at higher levels in orbital fibroblasts from inactive GO is required. Yet, our data strongly suggest that in the inactive stage of GO the orbital fibroblasts acquire effector functions that are strongly related to ECM remodeling.

### Proteome Pathway, Network and Functional Analysis

**Figure 2:**
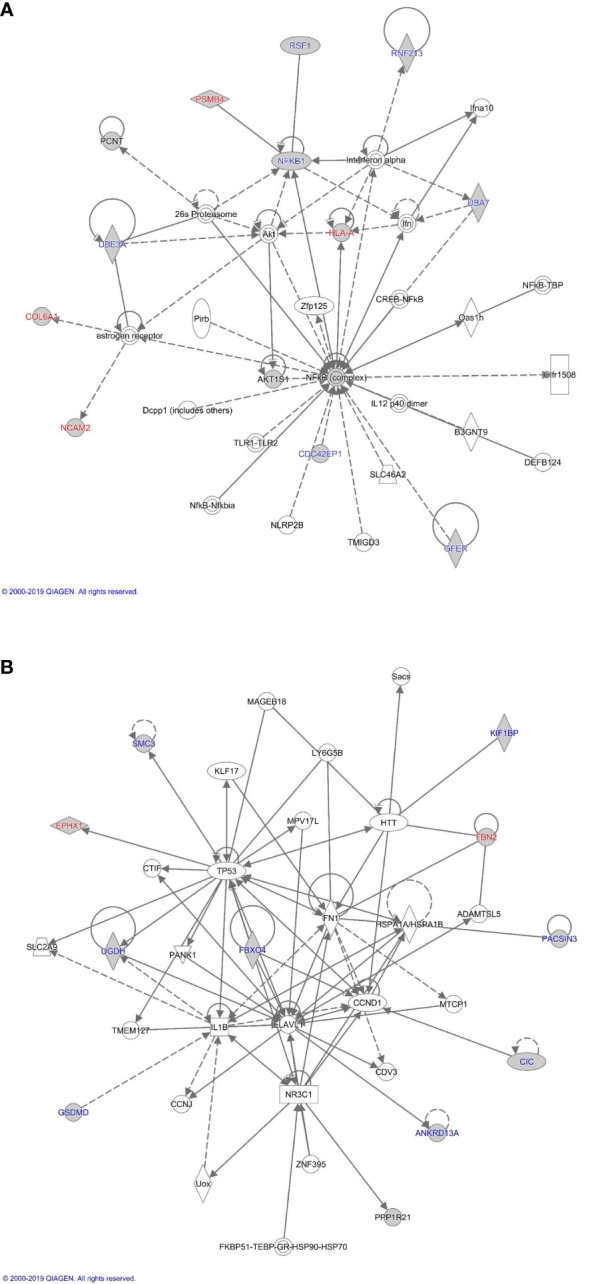
Proteome based network analysis. **(A)** Network 1: linked to cellular development and connective tissue disorders. Differentially expressed proteins are highlighted in grey. The differentially expressed proteins labelled in red were the up-regulated while differentially expressed proteins labelled in blue were down-regulated in inactive GO comparing to active GO orbital fibroblasts. **(B)** Network 2: linked to free radical scavenging, DNA replication, recombination and repair and cellular assembly and organization. Differentially expressed proteins are highlighted in grey. The differentially expressed proteins labelled in red were up-regulated while differentially expressed proteins labelled in blue were down-regulated in inactive GO comparing to active GO orbital fibroblasts.

The proteins differentially expressed between orbital fibroblasts from active GO and inactive GO were fed into Ingenuity with the aim to link these proteins to networks and pathways of interest to GO pathogenesis. The top two identified canonical pathways were UDP-D-xylose and UDP-D-glucuronate biosynthesis (p-value = 0.00217) and protein ubiquitination pathway (p-value = 0.00305) (**Table 2**). Moreover, all these proteins, except MT1X, were linked to 2 networks (**Figure 2**). The first network is associated with cellular development and connective tissue disorders (**Figure 2A**), while the second network is linked to free radical scavenging, DNA replication, recombination and repair and cellular assembly and organization (**Figure 2B**). In network 1, the differentially expressed proteins were linked with NF-κB, Akt, 26s proteasome, interferon alpha and estrogen receptor (**Figure 2A**). On the other hand, the differentially expressed proteins in network 2 were linked to several transcriptional regulators, such as cellular tumor antigen p53 (TP53), huntingtin (HTT), cyclin D1 (CCND1), and also other (pathogenic) proteins including fibronectin 1 (FN1) and Hsp70-binding protein 1 (HSPA) (**Figure 2B**).

**Table 2:** Top 5 canonical pathways and diseases and bio-functions from the differentially expressed proteins.

Top Canonical Pathways		
Name	p-value	Overlap
**UDP-D-xylose and UDP-D-glucuronate Biosynthesis**	0.00217	50.0%
**Protein Ubiquitination Pathway**	0.00305	1.1%
**Crosstalk between Dendritic Cells and Natural Killer Cells**	0.00418	2.2%
**Altered T Cell and B Cell Signaling in Rheumatoid Arthritis**	0.00427	2.2%
**OX40 Signaling Pathway**	0.00427	2.2%
**Top Diseases and Bio Functions**		
**Diseases and Disorders**		
**Name**	**p-value**	**#Molecules**
**Cancer**	0.00000287 – 0.049	24
**Endocrine System Disorders**	0.00000287 – 0.0436	24
**Organismal Injury and Abnormalities**	0.00000087 – 0.0498	24
**Reproductive System Disease**	0.0000428 – 0.0436	19
**Respiratory Disease**	0.0000551 – 0.0394	13
**Molecular and Cellular Functions**		
**Name**	**p-value**	**Proteins (listing from the lowest p-value)**
**Cell-To-Cell Signaling and Interaction**	0.000469 - 0.0434	HLA-A, NFKB1, UBE3A, CDC42EP1, NCAM2, SMC3
**Carbohydrate Metabolism**	0.00111 - 0.0445	UGDH, EPHX1, HLA-A, NFKB1
**Cell Death and Survival**	0.00111 - 0.0487	HLA-A, NFKB1, FBN2, PSMB4, GSDMD, SMC3, COL6A1, GFER, RSF1, AKT1S1, EPHX1, UBA7, UBE3A
**Cell Morphology**	0.00111 - 0.0434	NFKB1, PCNT, UBE3A, NCAM2, GFER, GSDMD, HLA-A, CDC42EP1
**Cellular Assembly and Organization**	0.00111 - 0.0424	KIF1BP, ANKRD13A, CDC42EP1, NCAM2, NFKB1, UBE3A, GFER, PCNT, RSF1, FBXO4, HLA-A

### Global DNA Methylation Profile in Orbital Fibroblasts

**Figure 3:**
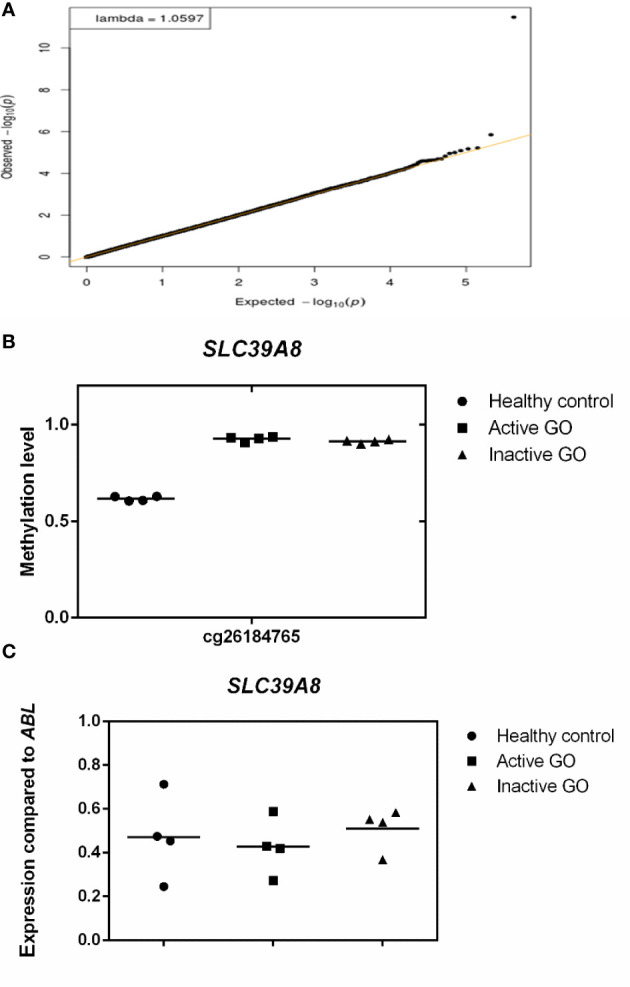
Comparison of orbital fibroblast global DNA methylation profiles with FDR <0.05. **(A)** DNA methylation in active GO orbital fibroblasts (n = 4), inactive GO orbital fibroblasts (n = 4) and healthy control orbital fibroblasts (n = 4) were compared. Global DNA methylation was measured using the Illumina Infinium Human Methylation 450K beadchip on bisulphite-treated DNA and analyzed by GenomeStudio methylation analysis package. Genes with differential DNA methylation were further corrected for multiple testing using FDR < 0.05. **(B)** Methylation level of *SLC39A8*. Methylation level ranges from 0 (unmethylated) to 1 (fully methylated). Individual symbols represent orbital fibroblast cultures from individual patients. Horizontal bar depicts the median. **(C)** *SLC39A8* expression was determined by real-time quantitative (RQ)-PCR and normalized to the control gene *ABL*. Data from mRNA expression was analyzed using ANOVA and subsequently analyzed with the Mann Whitney U test. Individual symbols represent orbital fibroblast cultures from individual patients. Horizontal bar depicts the median.

After adjustment for multiple testing using false discovery rate (FDR < 0.05), none of the genes were differentially methylated when comparing the orbital fibroblasts isolated from active GO (n = 4) and inactive GO patients (n = 4), although hypermethylated *GNAS* (cg09885502) showed a trend to reach statistical significance (FDR = 0.0774) in inactive GO (**Supplementary Figure 1**). However, GNAS was not found differentially expressed at the protein level with the current technique used (**Supplementary Figure 1**). *SLC39A8* was the only gene that showed statistically significant difference when comparing control orbital fibroblasts (n = 4) with all GO patients (active and inactive GO; n = 8) (FDR = 1.404 x 10^-6^) (**Figure 3A**), being hypermethylated in the 3’UTR region in the orbital fibroblasts from GO patients comparing to healthy control (**Figure 3B**). Although this could potentially be associated with decreased *SLC39A8* gene expression on GO orbital fibroblasts, no difference in mRNA expression was detected between the orbital fibroblast from inactive GO, active GO and controls (**Figure 3C**). Neither was SLC39A8 protein detected by our current proteomics approach. Gene expression level of enzymes regulating DNA methylation, the writers, DNA methyltransferases (DNMTs), and the erasers, ten eleven translocation (TET), were further examined; however, the expression level of all *DNMT* and *TET* were not significantly different in orbital fibroblasts from GO and healthy controls (**Supplementary Figure 2**).

### Hypermethylated Genes in Orbital Fibroblasts From Active GO Patients

**Figure 4:**
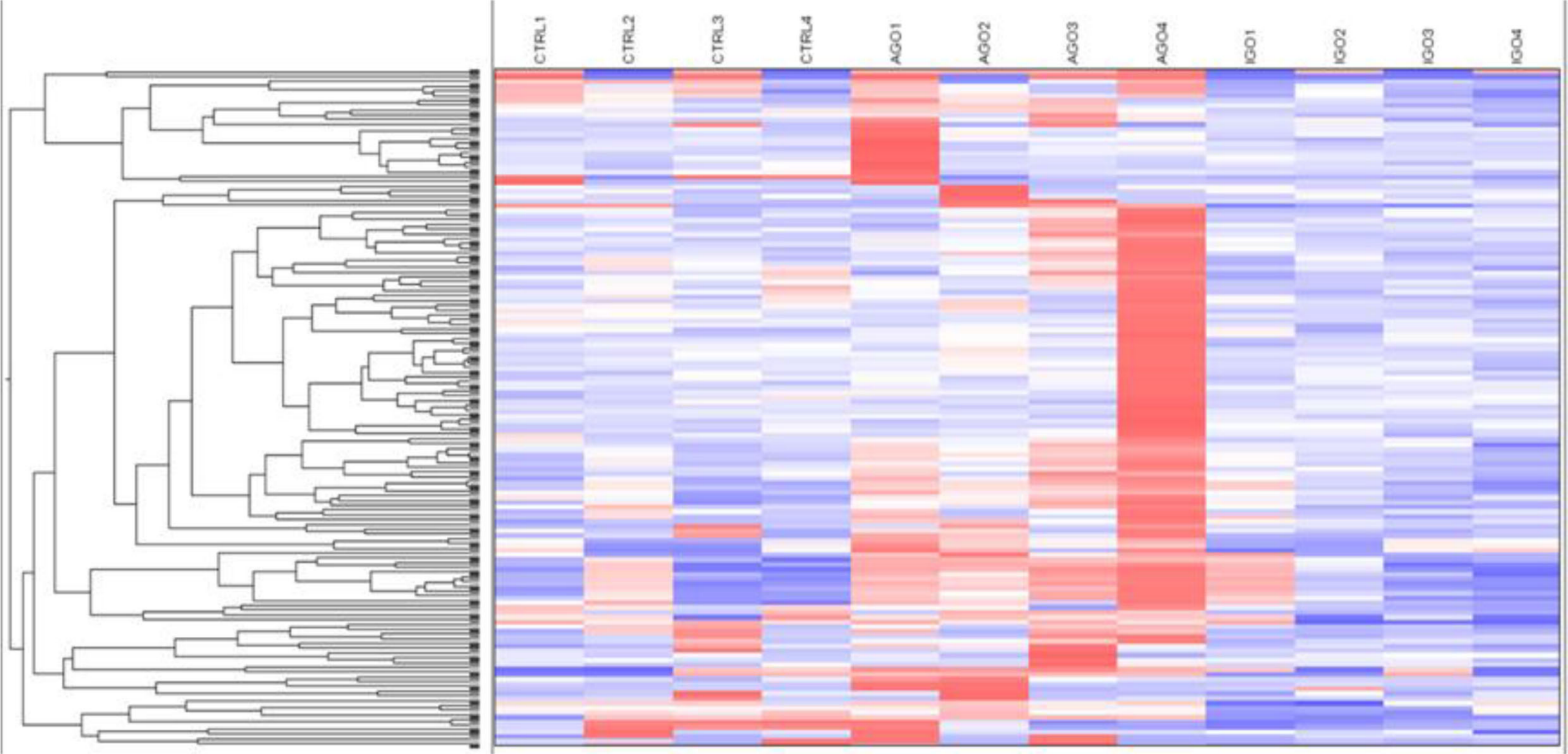
Hypermethylated genes in orbital fibroblasts from active GO in comparison to orbital fibroblasts from inactive GO and healthy controls with a fold difference ≥ 2. DNA methylation in active GO orbital fibroblasts (n = 4) was compared with inactive GO orbital fibroblasts (n = 4). Global DNA methylation was measured using the Illumina Infinium Human Methylation 450K beadchip on bisulphite-treated DNA and analyzed by GenomeStudio methylation analysis package. Hypermethylated genes with differences in DNA methylation level of at least 2-fold in active GO orbital fibroblasts were clustered as shown in the heatmap.

We further analyzed differences in methylation level in a less stringent manner by applying a cut-off difference of more than 2-fold between active and inactive GO patients. This resulted in a total of 142 hits, corresponding to 115 coding genes, that were hypermethylated in the orbital fibroblasts from active GO patients (**Figure 4** and **Supplementary Table 3**). Among the top 10 genes (with the fold differences ranging approximately from 3.1 to 6.9-fold), the highest fold difference was detected by a single probe located in the gene body of *WDR8* (**Supplementary Figure 3** and **Supplementary Table 3**). Several other genes were detected by more than one probe including *Mir548F5*, *MAB21L1*, *HTATIP2*, *TTC12*, *NIPAL2*, *TRIM2*, *PAQR5*, *OR2L13*, *RPH3AL*, *GPR6*, *MYOM2*, *DGKQ*, *ZNF234* and *SPAG1* (**Supplementary Figure 3**).

### Hypomethylated Genes in Orbital Fibroblasts From Active GO Patients

**Figure 5:**
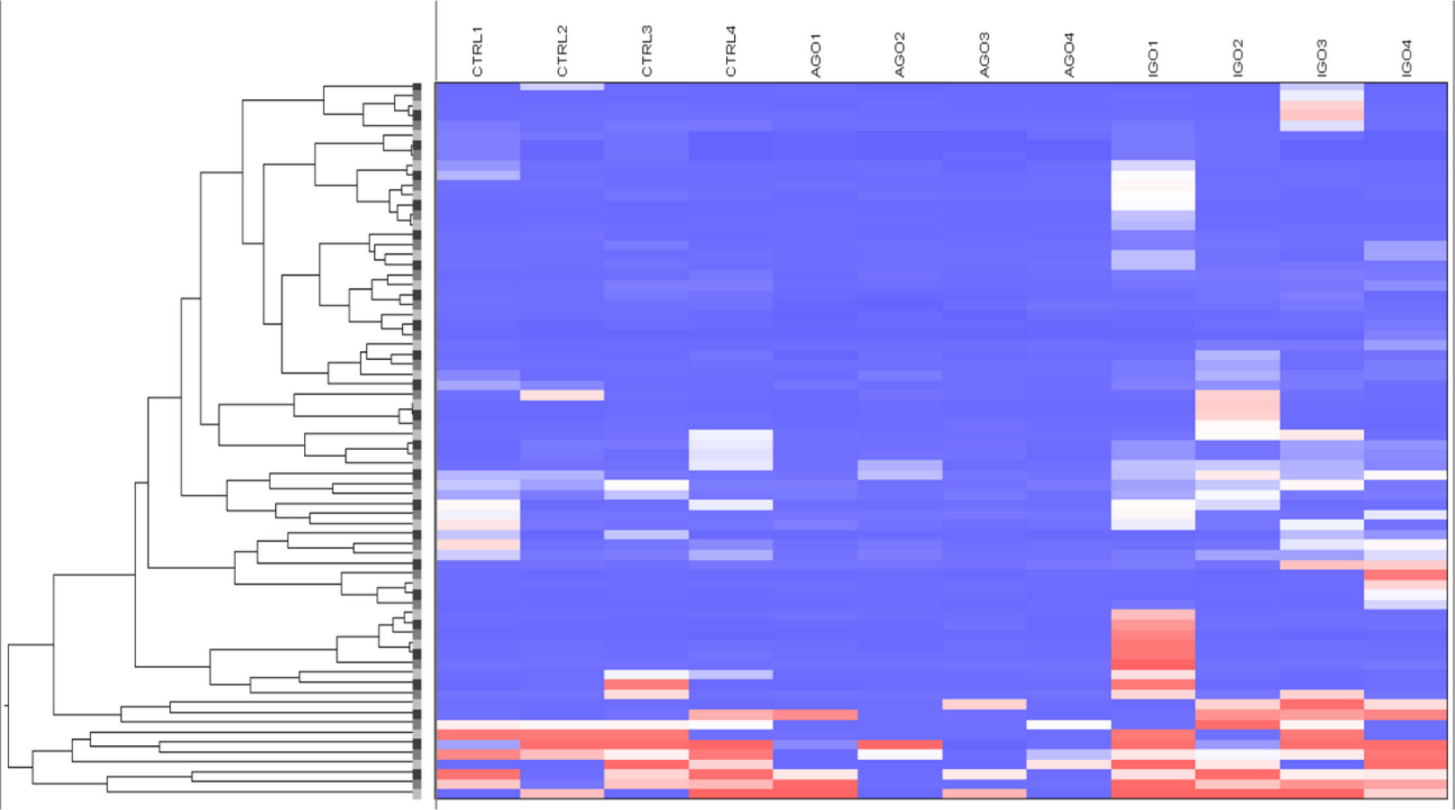
Hypomethylated genes in orbital fibroblasts from active GO in comparison to orbital fibroblasts from inactive GO and healthy controls with a fold difference ≥ 2. DNA methylation in active GO orbital fibroblasts (n = 4) were compared with inactive GO orbital fibroblasts (n = 4). Global DNA methylation was measured using the Illumina Infinium Human Methylation 450K beadchip on bisulphite-treated DNA and analyzed by GenomeStudio methylation analysis package. Hypomethylated genes with differences in DNA methylation level of at least 2-fold in active GO orbital fibroblasts were clustered as shown in the heatmap.

**Figure 6:**
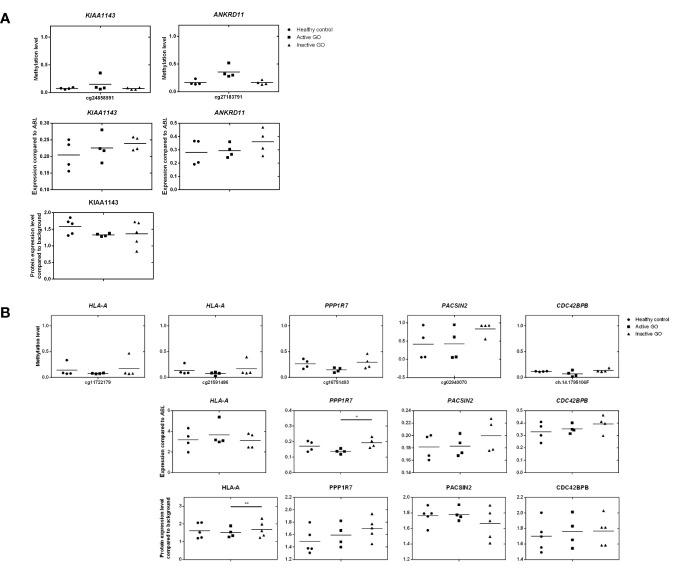
Comparison of DNA methylation, mRNA expression and proteomic data. **(A)** Comparison of hypermethylated genes, mRNA expression and proteomic data. Gene expression was determined by real-time quantitative (RQ)-PCR and normalized to the control gene *ABL*. Data from mRNA expression was analyzed using ANOVA and subsequently analyzed with the Mann Whitney U test. Individual symbols represent orbital fibroblast cultures from individual patients. Horizontal bar depicts the median. **(B)** Comparison of hypomethylated genes, mRNA expression and proteomic data. Gene expression was determined by real-time quantitative (RQ)-PCR and normalized to the control gene *ABL*. Data from mRNA expression was analyzed using ANOVA and subsequently analyzed with the Mann Whitney U test. Individual symbols represent orbital fibroblast cultures from individual patients. Horizontal bar depicts the median.

Applying the cut-off difference of more than 2-fold between active and inactive GO patients yielded 66 hits, corresponding for 63 genes, that were hypomethylated in the orbital fibroblasts from active GO compared with orbital fibroblasts from inactive GO patients (**Figure 5** and **Supplementary Table 4**). The gene set hypomethylated in active GO orbital fibroblasts comprised a.o.: *RNF168* (with the highest fold change of 5.08-fold, as detected by one probe in the gene body), *SIM2* (detected with 2 different probes located in the gene body: 2.19-fold differences at cg15316660 and 2.03-fold differences at cg23286646 in the gene body), *AIRE* (detected with 2 different probes: 2.35-fold difference at cg27251412 and 2.34-fold difference at cg09510531 200 nucleotides upstream of the transcriptional start site; TSS200) and *HLA-A* (detected with 2 different probes: 2.27-fold difference at cg11722179 and 2.22-fold difference at cg21591486 in the gene body) (**Figure 6B** and **Supplementary Figure 4**).

To investigate the effects of differential methylation at the TSS200 in *AIRE* and gene-body in *HLA-A*, mRNA expression levels were determined by RQ-PCR. *AIRE* gene expression was not detectable by RQ-PCR in any of the orbital fibroblasts (data not shown), while *HLA-A* was not differentially expressed at the mRNA level between fibroblasts from active GO and inactive GO (**Figure 6B**). AIRE protein expression was not detected by the current proteomics approach (data not shown), while HLA-A protein expression was significantly down-regulated in active GO orbital fibroblasts (**Figure 6B**).

### Integrative Analysis of Proteomics and Gene Methylation in Orbital Fibroblasts From Active GO Patients

Upon integration of the DNA hypermethylation data with the proteome data, *KIAA* and the *ANKRD* gene family emerged from both types of analyses and were therefore further examined by RQ-PCR. Although hypermethylation of *KIAA1143* and *ANKRD11* genes was more than 2-fold, neither *KIAA1143* nor *ANKRD11* mRNA expression reached statistically significant difference in expression when comparing active and inactive GO orbital fibroblasts (**Figure 6A**). In addition, ANKRD11 protein was not detected by the current proteomics approach (data not shown). Neither the difference on gene expression data and proteome data reached statistical significance nor corresponded to the DNA methylation data.

Integration of the DNA hypermethylation data with the proteome data revealed overlap for PPP1R, PACSIN, and the CDC42 family, which were subsequently further validated by RQ-PCR. Although trends towards elevated mRNA expression for *PACSIN2* and *CDC42BPB* were observed in inactive GO, no statistical significance existed among orbital fibroblast groups, while *PPP1R7* mRNA expression was significantly (p < 0.05) higher in inactive GO orbital fibroblasts than in active GO orbital fibroblasts (P < 0.05) (**Figure 6B**). However, neither the mRNA expression data nor the proteome data corresponded to the DNA methylation data.

### Network Analysis From Methylated Genes in Orbital Fibroblasts From GO Patients

**Figure 7:**
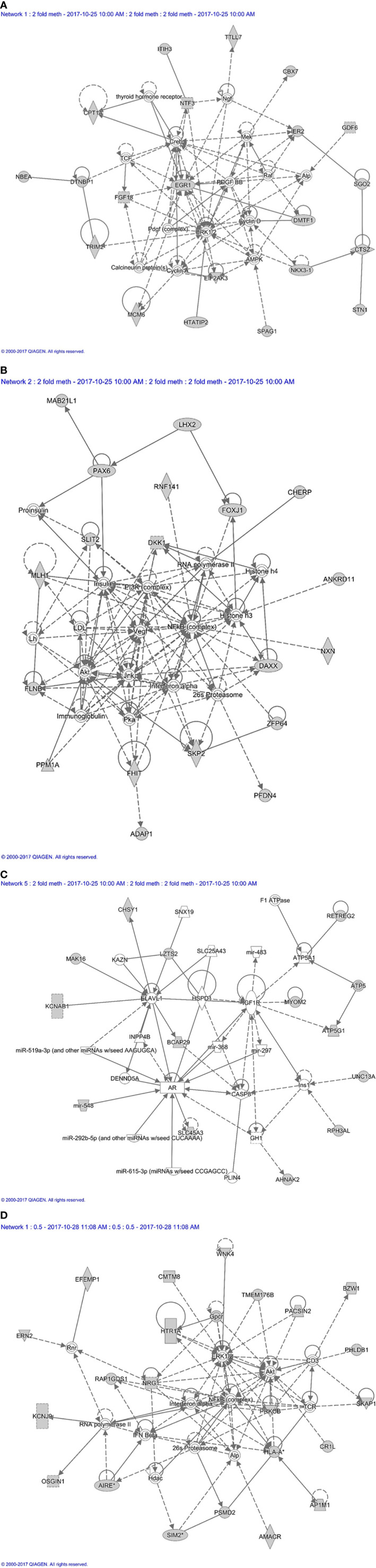
Methylation based network analysis. **(A)** Network 1 derived from hypermethylated genes in orbital fibroblasts from active GO: linked to neurological disease, organismal injury and abnormalities, and nervous system development and function Network analysis was further performed on these differentially hypermethylated genes (highlighted in grey) using Ingenuity (Qiagen) filtering only for those networks and pathways that are based on experimentally obtained information. **(B)** Network 2 derived from hypermethylated genes in orbital fibroblasts from active GO: linked to embryogenic development, nervous system development and function, and organ development. Network analysis was further performed on these differentially hypermethylated genes (highlighted in grey) using Ingenuity (Qiagen) filtering only for those networks and pathways that are based on experimentally obtained information. **(C)** Network 3 derived from hypermethylated genes in orbital fibroblasts from active GO: linked to cardiovascular system development and function, organismal development, and cellular assembly and organization. Network analysis was further performed on these differentially hypermethylated genes (highlighted in grey) using Ingenuity (Qiagen) filtering only for those networks and pathways that are based on experimentally obtained information. **(D)** Network 4 derived from hypomethylated genes in orbital fibroblasts from active GO: linked to cellular growth and proliferation, nervous system development and function, and cell-to-cell signaling and interaction. Network analysis was further performed on these differentially hypomethylated genes (highlighted in grey) using Ingenuity (Qiagen) filtering only for those networks and pathways that are based on experimentally obtained information.

Next, all the genes that displayed differential DNA methylation between orbital fibroblasts from active GO and inactive GO were fed into Ingenuity pathway analysis to identify potential critical molecular networks involved in GO pathogenesis. The top network with the highest score obtained for hypermethylated genes in active GO orbital fibroblasts genes included TSHR signaling, PDGF-BB signaling and the ERK1/2 signal transduction pathway, as depicted in network 1 (**Figure 7A**). Ingenuity linked this network mainly to neurological disease, organismal injury and abnormalities, and nervous system development and function. *EGR1*, one of the top 10 genes with the highest fold methylation difference, was also found in this network. The second network with the second highest score (designated as network 2) contained genes that were associated with the PI3K, NF-κB, JNK and Akt signaling pathways (**Figure 7B**). Ingenuity linked this network mainly to embryogenic development, nervous system development and function, and organ development. Clearly, these signaling cascades exert several important roles in immune activation as well as fibrosis (27, 44, 50, 51). In addition, *Mir548F5* was found in another network (network 3) together with *MYOM2* and *RPH3AL* and were connected to IGF-1R, androgen receptor and various non-coding RNAs (**Figure 7C**). Ingenuity linked this network mainly to cardiovascular system development and function, organismal development, and cellular assembly and organization. Moreover, *LZTS2* and *MYOM2* found in this network are among the top 10 genes with the highest fold difference in methylation status (**Figure 7C**).

For hypomethylated genes in active GO orbital fibroblasts, 6 of the top 10 genes with the highest fold difference were found in network 4, including *ERN2*, *CMTM8*, *BZW1*, *PRKCB*, *PSMD2* and *AP1M1* (**Figure 7D**). Of the genes that were found hypomethylated in orbital fibroblasts from active GO, *AIRE* and *SIM2* linked to network (network 4) that also contained NF-κB, ERK1/2 and Akt signaling pathways (**Figure 7D**). *HLA-A* and *PSMD2* (proteasome 26S subunit, non-ATPase 2), that are associated with peptide degradation, antigen presentation and TCR signaling processes, also linked to network 4 (**Figure 7D**). Ingenuity associated network 4 mainly with cellular growth and proliferation, nervous system development and function, and cell-to-cell signaling and interaction.

### Pathway and Functional Analysis From Methylated Genes in the Orbital Fibroblasts From GO Patients

The top 5 canonical pathways obtained from genes hypermethylated in orbital fibroblasts from active GO are shown in **Table 3**, while for genes hypomethylated in orbital fibroblasts form active GO the top 5 canonical pathways are given in **Table 4**. When all the functions from genes hypermethylated and hypomethylated in active GO (**Tables 3** and **4**) were integrated, overlapping bio-functions and diseases became apparent. The top disease/disorder identified to be shared between the hypo- and hypermethylated genes was organismal injury and abnormalities, containing ninety-seven hypermethylated genes (p-value = 1x10^-6^ – 0.0145) and sixty-two hypomethylated genes (p-value = 9.88x10^-5^ – 0.0498). For the molecular and cellular functions, cellular development and cellular growth and proliferation were among the top molecular and cellular functions that were shared between the hypo- and hypermethylated genes. Cellular development linked to 28 hypermethylated genes (p-value = 7.02x10^-5^ – 0.0145) (**Table 3**) and nineteen hypomethylated genes (p-value = 0.00132 – 0.0469) **(** **Table 4**), while cellular growth and proliferation linked to twenty-five hypermethylated genes (p-value = 7.02x10^-5^ – 0.0145) (**Table 3**) and seventeen hypomethylated genes (p-value = 1.32x10^-4^ – 0.0498) (**Table 4**).

**Table 3:** Top 5 canonical pathways and diseases and bio-functions from the hypermethylated genes with the cut-off at more than 2-fold difference in the active GO orbital fibroblasts.

Top Canonical Pathways		
Name	p-value	Overlap
**Tyrosine Biosynthesis IV**	0.0145	33.3%
**Phenylalanine Degradation I (Aerobic)**	0.0193	25.0%
**Chondroitin and Dermatan Biosynthesis**	0.0289	16.7%
**Glutamate Receptor Signaling**	0.0315	3.5%
**NAD Biosynthesis from 2-amino-3-carboxymuconate Semialdehyde**	0.0336	14.3%
**Top Diseases and Bio Functions**		
**Diseases and Disorders**		
**Name**	**p-value**	**#Molecules**
**Cancer**	1x10^-6^ – 0.0145	95
**Gastrointestinal Disease**	1x10^-6^ – 0.0145	90
**Organismal Injury and Abnormalities**	1x10^-6^ – 0.0145	97
**Developmental Disorder**	2.22x10^-5^ – 0.0145	26
**Ophthalmic Disease**	2.22x10^-5^ – 0.0145	9
**Molecular and Cellular Functions**		
**Name**	**p-value**	**#Molecules**
**Gene Expression**	7.0x10^-5^ – 0.00209	30
**Cellular Development**	7.0x10^-5^ – 0.0145	28
**Cellular Growth and Proliferation**	7.0x10^-5^ – 0.0145	25
**Cell Cycle**	4.85x10^-4^ – 0.0145	18
**Cell Morphology**	4.85x10^-4^ – 0.0145	14

**Table 4:** Top 5 canonical pathways and diseases and bio-functions from the hypomethylated genes with the cut-off at more than 2-fold difference in the active GO orbital fibroblasts.

Top Canonical Pathways		
Name	p-value	Overlap
**Lipoate Salvage and Modification**	0.003	100.0%
**Lipoate Biosynthesis and Incorporation II**	0.00598	50.0%
**Dopamine-DARPP32 Feedback in cAMP Signaling**	0.0132	1.8%
**Nur77 Signaling in T Lymphocytes**	0.0136	3.4%
**Wnt/Ca^2+^ pathway**	0.0154	3.2%
**Top Diseases and Bio Functions**		
**Diseases and Disorders**		
**Name**	**p-value**	**#Molecules**
**Cancer**	9.88x10^-5^ – 0.0498	61
**Organismal Injury and Abnormalities**	9.88x10^-5^ – 0.0498	62
**Dermatological Diseases and Conditions**	4.73x10^-4^ – 0.0496	43
**Gastrointestinal Disease**	0.00106 – 0.044	55
**Metabolic Disease**	0.00172 – 0.0498	8
**Molecular and Cellular Functions**		
**Name**	**p-value**	**#Molecules**
**Cellular Growth and Proliferation**	1.32x10^-4^ – 0.0498	17
**Cellular Movement**	0.00117 – 0.0498	12
**Cell-To-Cell Signaling and Interaction**	0.00132 – 0.0498	13
**Cellular Development**	0.00132 – 0.0469	19
**Drug Metabolism**	0.00132 – 0.0452	4

## Discussion

Disease activity and characteristics of GO alter with disease progression. Early active GO is characterized by active inflammation that progresses into a late inactive phase without apparent inflammation but characterized by extensive orbital tissue remodeling (increased adipose tissue formation, excessive hyaluronan deposition and fibrosis). Orbital fibroblasts represent the main effector cells in GO and contribute to both the early active and late inactive phases of disease (8). Here, we performed extensive proteomics and global DNA methylation analysis of orbital fibroblasts from active and inactive GO as well as control individuals. Our data clearly demonstrate distinctive proteome and DNA methylation profiles between orbital fibroblasts from patients with active and inactive GO. These profiles can be linked to pathologically relevant molecular networks specifically associated with active/inactive disease stage, including inflammation, cellular development, growth, proliferation and tissue remodeling. Therefore, this data clearly illustrates that with GO disease progression orbital fibroblasts exhibit specific characteristics linked to disease stage specific pathology, and that epigenetic regulation is involved in controlling this.

Orbital fibroblasts from active GO displayed overexpression of proteins typically involved in inflammation, cellular proliferation, hyaluronan synthesis and adipogenesis (**Figure 1C**). In contrast, various proteins linked to ECM biology and fibrotic disease were overexpressed in orbital fibroblasts from inactive GO (**Figure 1B**). These findings corroborated with the different types of networks identified, which included cellular development and connective tissue disorders, DNA replication, recombination and repair and cellular assembly and organization (**Figure 2**). Although our current protein findings fit the pathophysiological model of GO, further studies to unravel the exact function of these proteins in orbital fibroblasts as well as their contribution to the different stages of GO are required.

To our knowledge, our study is the first that explored DNA methylation in orbital fibroblasts from GO patients. Comparison of DNA methylation with stringent analysis using FDR between healthy controls and all the GO orbital fibroblasts included in our study resulted in a significant difference in methylation only for *SLC39A8* (hypermethylation in 3’UTR region in case of the GO orbital fibroblasts; **Figure 3**). SLC39A8 is a member of the major zinc transporter SLC39 (ZIP) family, and cellular zinc import by SLC39A8 is crucial in the control of immune activation and function (52, 53). SLC39A8 methylation status did not correlate with mRNA expression, while SLC39A8 protein was not detected at all. However, the SLC93A8 hypermethylation in GO orbital fibroblasts located to the 3’UTR region, a region in which hypermethylation does not necessarily relate to transcriptional repression as commonly reported in promoter regions (54–56). We can, however, not exclude that SLC39A8 expression is also controlled by other regulatory mechanism such as small non-coding RNAs. SLC39A8 is also thought to be the primary transporter of the toxic cation cadmium, which is found in cigarette smoke. In this study we did not detect SLC93A8 protein, which might be related to the sensitivity of the technique we applied. Yet, we propose that additional studies to understand the role of the SLC39A8 and other SLC39 family of solute carriers in the pathogenesis of GO are potentially of great interest considering that smoking is a major environmental risk factor for GO (57).

Additional analysis of global DNA methylation using a 2-fold difference cut-off was conducted with the intend to lower the stringency from FDR analysis. A drawback of this approach is however that individual outliers have a relatively large effect on the final outcome, which either may mask potential important differences in methylation or identify false positive methylation differences, especially with the small groups (n = 4 per group) used in this study. Nonetheless, the overall DNA methylation patterns we observed suggest that orbital fibroblasts from inactive GO are more comparable to control orbital fibroblasts than to orbital fibroblasts from active GO (**Figures 4** and **5**). The global DNA methylation patterns and associated networks we found corroborate with the networks generated from the proteomics analysis, including NF-κB, Akt and IFNα (**Figures 2** and **7**), as well as previous reports that linked these pathways to GO pathogenesis (58–60). Other genes hypermethylated in inactive GO orbital fibroblasts also linked to other pathogenic pathways previously reported in GO, including PDGF (44), thyroid hormone receptor (61), NGF (62), AMPK (63) and ERK1/2 signaling pathways (64, 65) (**Figure 7**). In orbital fibroblasts from active GO hypermethylated genes clearly linked to inflammation, while hypomethylated genes linked to adipogenesis and autoimmune-related genes. This is in line with the association to inflammation and adipogenesis that we observed for the proteome of active GO orbital fibroblasts.

Genes hypomethylated in active GO orbital fibroblasts included *AIRE* and *HLA-A* (**Figure 6B** and **Supplementary Figure 4**). *AIRE* controls tissue specific antigen expression by medullary thymic epithelial cells to control thymic T-cell selection, and AIRE deficiency is associated with autoimmune disease (66, 67). CpG methylation in the promoter of the *AIRE* gene has previously been reported to control its tissue-specific expression pattern (68). Although hypomethylation of *AIRE* promoter was found in orbital fibroblasts from active GO (**Supplementary Figure 4**), *AIRE* expression was undetectable at mRNA and protein level (data not shown). Several studies showed *AIRE* expression in fibrocytes that typically infiltrate the orbital tissue form GO patients (69, 70). Potentially, fibrocyte numbers in our heterogeneous orbital fibroblast populations were low, which did however still allow detection of differences in *AIRE* methylation but not in AIRE mRNA or protein. *HLA-A* hypomethylation was detected in the gene body in orbital fibroblasts from active GO (**Figure 6B**). While *HLA-A* mRNA expression level did not differ between active and inactive GO, HLA-A protein expression was significantly down-regulated in active GO orbital fibroblasts (**Figure 6B**). Decreased HLA-A protein expression as observed in the orbital fibroblasts from active GO might regulate antigen presentation by these cells.

Fibroblasts isolated from fibrotic tissue display phenotypes that remain stable in *in vitro* culture for prolonged periods of time (71, 72). This allows investigation of epigenetic alterations and regulation, including histone modification, DNA methylation and non-coding RNA (71, 73). In general, we observed a poor correlation between protein expression, DNA methylation and mRNA expression. Poor correlations between DNA promotor methylation level and gene expression, as well as both positive and negative correlations between DNA gene body methylation and gene expression, have been reported (74–76). Our study thus clearly indicates that the proteome alterations we observed in orbital fibroblasts in relation to the specific GO disease stage are controlled by regulatory layers additional to DNA methylation (e.g. histone modifications and small regulatory RNA molecules) that remain so-far hardly studied in GO. For example, several microRNAs, including miR-322, miR-508-3p, miR-9 and miR-16 have been described to target *NFKB1* mRNA (77–80). miR-16 was previously found to be upregulated in orbital tissue from inactive GO patients and might thus be involved in downregulating NFKB1 and resolving inflammation that occurs during this disease stage (81). Altogether, the current data support the role of NF-κB signaling in regulating inflammation in GO orbital fibroblasts. Moreover, the data point towards existence of a complex epigenetic regulation machinery that controls NFKB1 expression and activity with GO disease progression.

**Figure 8:**
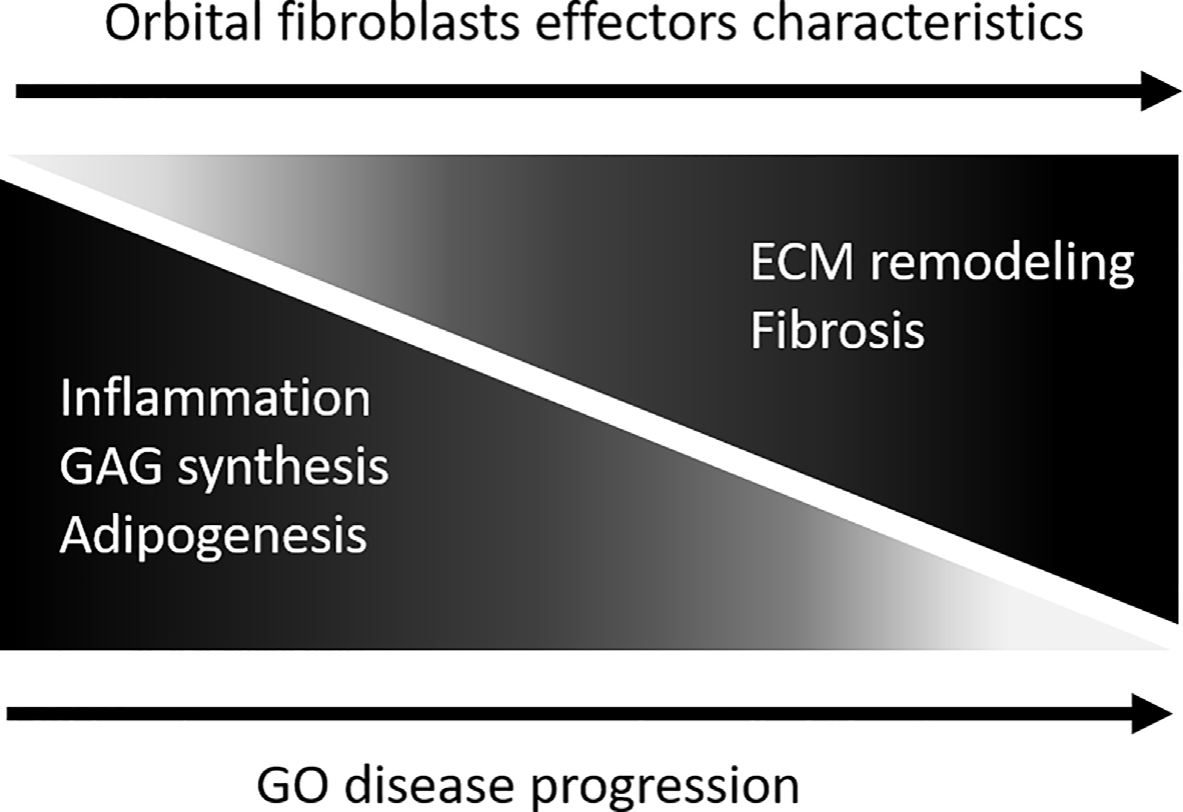
Model depicting orbital fibroblast effector characteristics upon GO disease progression. During active disease orbital fibroblasts are skewed towards a functional phenotype involved in in inflammation, glycosaminoglycan synthesis and adipogenesis. With disease progression the orbital fibroblasts undergo a switch from this “inflammatory/pro-adipogenic primed” effector cell towards a “remodelling/pro-fibrotic” type of effector cell.

There are several limitations associated with our study. First, the sample size is small, and it cannot be excluded that the extended orbital fibroblast isolation protocol and culture procedure altered the *in vivo* phenotypic/effector cell character of the orbital fibroblasts, although long term (epigenetic)stability of fibroblasts in culture is previously described (71, 72). Second, orbital fibroblasts are heterogeneous (8). Therefore, use of purified fibroblast subpopulations on the basis of cell surface marker expression, for instance Thy-1, CD34 and TSHR, could generate more in-depth insight into epigenetic regulation and fibroblast-subtype specific proteomes in GO. Despite these limitations, our proteomic and DNA methylation analysis identified known as well as novel molecules and molecular networks in relation to the pathogenesis of GO. In addition, both the proteomics and DNA methylation support that, with GO disease progression, orbital fibroblasts undergo a switch from an “inflammatory/pro-adipogenic primed” effector cell to a “remodeling/pro-fibrotic” type of effector cell, as depicted in **Figure 8**. We propose that detailed molecular understanding of this “inflammatory/pro-angiogenic-to-remodeling/pro-fibrotic switch” should be an active field of future research as it can contribute to improvement of treatment protocols.

## Data Availability Statement

The mass spectrometry proteomics data have been deposited to the ProteomeXchange Consortium *via* the PRIDE partner repository with the dataset identifier PXD022257.

## Ethics Statement

The studies involving human participants were reviewed and approved by protocol ID-2007-01. The patients/participants provided their written informed consent to participate in this study.

## Author Contributions

SV designed research, performed research, analyzed and interpreted data, wrote the paper. PS performed research, analyzed data, wrote the paper. TPi designed research, analyzed data, contributed analytic tools, prepared manuscript. PS analyzed data, contributed analytic tools. DP contributed patient materials. VD, PH, NH, and TPa analyzed data, prepared manuscript. WD designed research, analyzed and interpreted data, prepared manuscript. All authors contributed to the article and approved the submitted version.

## Funding

This research was funded by the TSRI Fund (CU_FRB640001_01_23_1), Ratchadapisek Sompoch Endowment Fund (2017), Chulalongkorn University (760001-HR), Asia Research Center, Chulalongkorn University (007/2560) and Chulalongkorn University Office of International Affairs Scholarship for Short-term Research (3/2561).

## Conflict of Interest

The authors declare that the research was conducted in the absence of any commercial or financial relationships that could be construed as a potential conflict of interest.

## Supplementary Material

Supplementary Figure 1 Comparison of GNAS DNA methylation and protein expression data.

Supplementary Figure 2 Expression of *DNMT* and *TET* genes were determined by real-time quantitative (RQ)-PCR and normalized to the control gene *ABL*. Individual symbols represent orbital fibroblast cultures from individual patients. Horizontal bar depicts the median.

Supplementary Figure 3 Methylation level of hypermethylated genes in orbital fibroblasts from active GO. Methylation level ranges from 0 (unmethylated) to 1 (fully methylated). Genes that were detected with the highest fold difference between active and inactive GO orbital fibroblasts, *WDR8*, and genes that were detected by more than one probe, *Mir548F5* and *MAB21L1*, are shown in this figure. Individual symbols represent orbital fibroblast cultures from individual patients. Horizontal bar depicts the median.

Supplementary Figure 4 Methylation level of hypomethylated genes in orbital fibroblasts from active GO. Methylation level ranges from 0 (unmethylated) to 1 (fully methylated). Genes that were detected with the highest fold difference between active and inactive GO orbital fibroblasts, *RNF168*, and genes that were detected by more than one probe, *AIRE* and *SIM2*, are shown in this figure. Individual symbols represent orbital fibroblast cultures from individual patients. Horizontal bar depicts the median.
