# Supplementary figures and images for: Integrative Analysis of Proteomics and DNA Methylation in Orbital Fibroblasts From Graves’ Ophthalmopathy
Source: Front Endocrinol (Lausanne). 2021 Feb 15;11:619989. doi: 10.3389/fendo.2020.619989 (PMC7919747; doi:10.3389/fendo.2020.619989)

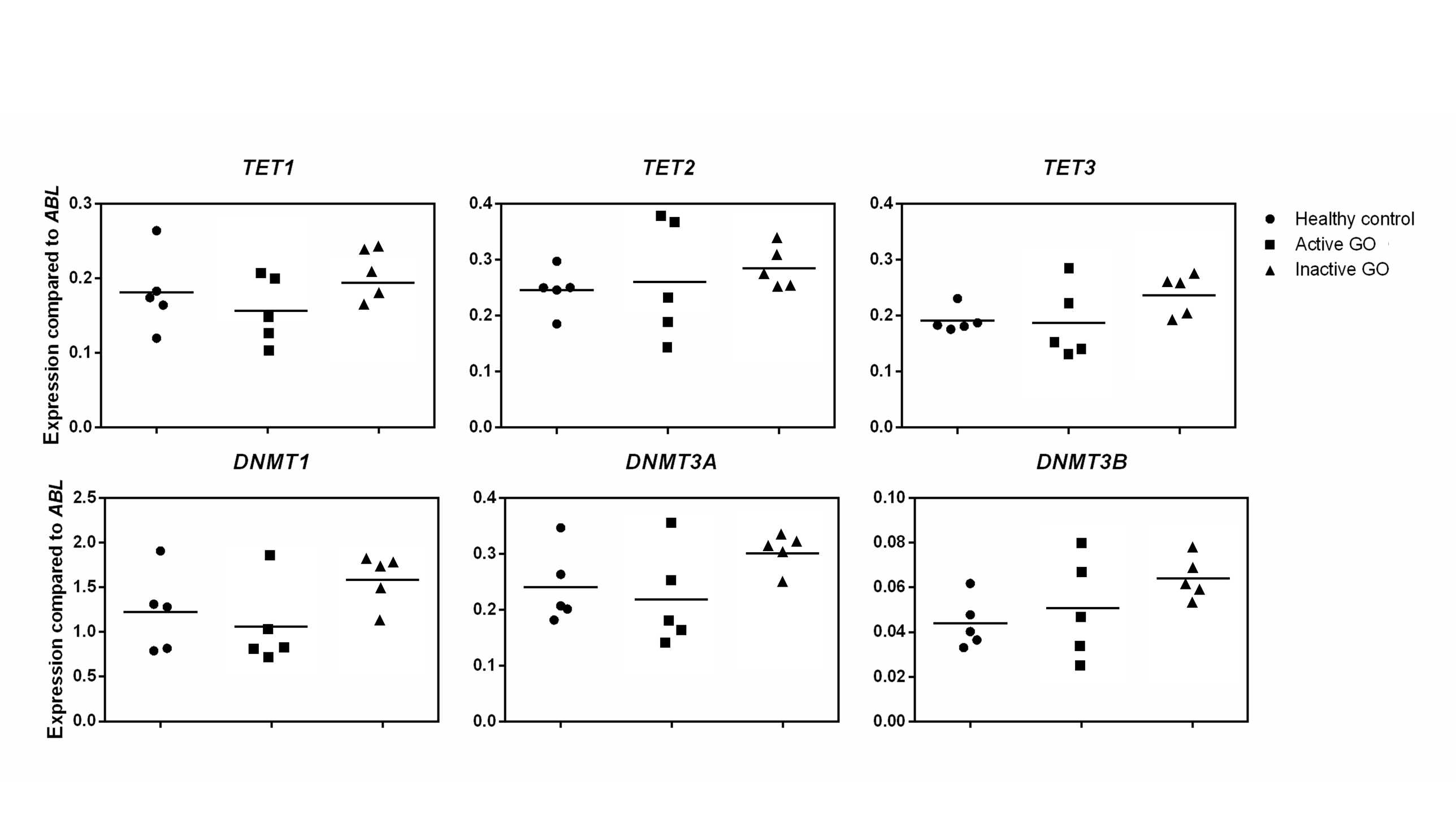

Supplement: Supplementary Figure 2 — Expression of DNMT and TET genes were determined by real-time quantitative (RQ)-PCR and normalized to the control gene ABL. Individual symbols represent orbital fibroblast cultures from individual patients. Horizontal bar depicts the median. [file Image_2.tif]
