# Supplementary material for: Integrative Analysis of Proteomics and DNA Methylation in Orbital Fibroblasts From Graves’ Ophthalmopathy
Source: Front Endocrinol (Lausanne). 2021 Feb 15;11:619989. doi: 10.3389/fendo.2020.619989 (PMC7919747; doi:10.3389/fendo.2020.619989)
Supplement: Supplementary file 5 [file Table_1.docx]

**Supplementary table 1** Primer-probe combinations used from TaqMan assay

| Gene | Forward primer (5’– 3’) | Reverse primer (5’– 3’) | Probe (5’FAM – 3’TAMRA) |
| --- | --- | --- | --- |
| *ABL* | TGGAGATAACATCTAAGCATAACTAAAGGT | GATGTAGTTGCTTGGGACCCA | CCATTTTTGGTTTGGGCTTCACACCATT |
| *PSMB4* | TaqMan Gene Expression Assays (Hs00160598_m1), Life technologies, Foster, CA. | | |
| *FBN2* | TaqMan Gene Expression Assays (Hs00266592_m1), Life technologies. | | |
| *COL6A1* | TaqMan Gene Expression Assays (Hs01095585_m1), Life technologies. | | |
| *NCAM2* | TaqMan Gene Expression Assays (Hs01562296_m1), Life technologies. | | |
| *PACSIN3* | TaqMan Gene Expression Assays (Hs00367625_m1), Life technologies. | | |
| *NFKB1* | TaqMan Gene Expression Assays (Hs00765730_m1), Life technologies. | | |
| *SMC3* | TaqMan Gene Expression Assays (Hs00271322_m1), Life technologies. | | |
| *GFER* | TaqMan Gene Expression Assays (Hs00193365_m1), Life technologies. | | |
| *GSDMD* | TaqMan Gene Expression Assays (Hs00986739_g1), Life technologies. | | |
| *UGDH* | TaqMan Gene Expression Assays (Hs01097550_m1), Life technologies. | | |
| *MT1X* | TaqMan Gene Expression Assays (Hs00745167_sH), Life technologies. | | |
| *SLC39A8* | TaqMan Gene Expression Assays (Hs00223357_m1), Life technologies. | | |
|  |  | | |
| *KIAA1143* | TaqMan Gene Expression Assays (Hs05634467_g1), Life technologies. | | |
| *ANKRD11* | TaqMan Gene Expression Assays (Hs00946580_m1), Life technologies. | | |
| *HLA-A* | TaqMan Gene Expression Assays (Hs01058806_g1), Life technologies. | | |
| *PPP1R7* | TaqMan Gene Expression Assays (Hs00160366_m1), Life technologies. | | |
| *PACSIN2* | TaqMan Gene Expression Assays (Hs01060727_m1), Life technologies. | | |
| *CDC42BPB* | TaqMan Gene Expression Assays (Hs00998688_m1), Life technologies. | | |
| *DNMT1* | TaqMan Gene Expression Assays (Hs00154749_m1), Life technologies. | | |
| *DNMT3A* | TaqMan Gene Expression Assays (Hs01027166_m1), Life technologies. | | |
| *DNMT3B* | TaqMan Gene Expression Assays (Hs00171876_m1), Life technologies. | | |
| *TET1* | TaqMan Gene Expression Assays (Hs00286756_m1), Life technologies. | | |
| *TET2* | TaqMan Gene Expression Assays (Hs00325999_m1), Life technologies. | | |
| *TET3* | TaqMan Gene Expression Assays (Hs00379125_m1), Life technologies. | | |
