# Supplementary material for: Integrative Analysis of Proteomics and DNA Methylation in Orbital Fibroblasts From Graves’ Ophthalmopathy
Source: Front Endocrinol (Lausanne). 2021 Feb 15;11:619989. doi: 10.3389/fendo.2020.619989 (PMC7919747; doi:10.3389/fendo.2020.619989)
Supplement: Supplementary file 6 [file Table_2.docx]

**Supplementary table 2** List of proteins with differential expression in the orbital fibroblasts from active and inactive GO (FDR < 0.05).

| **Gene** | **p-value** |
| --- | --- |
| ***SMC3*** | 0.005468 |
| ***HLA-A*** | 0.007962 |
| ***ANKRD13A*** | 0.008015 |
| ***PSMB4*** | 0.010799 |
| ***RSF1*** | 0.01721 |
| ***AKT1S1*** | 0.017784 |
| ***FBXO4*** | 0.02108 |
| ***NCAM2*** | 0.027867 |
| ***NFKB1*** | 0.028348 |
| ***KIAA1279*** | 0.029004 |
| ***FBN2*** | 0.02994 |
| ***COL6A1*** | 0.031695 |
| ***GSDMD*** | 0.032252 |
| ***EPHX1*** | 0.032258 |
| ***CDC42EP1*** | 0.035727 |
| ***PACSIN3*** | 0.039287 |
| ***UGDH*** | 0.039878 |
| ***MT1X;MT1G;MT2A*** | 0.041842 |
| ***RNF213*** | 0.041942 |
| ***UBE3A*** | 0.04229 |
| ***GFER*** | 0.04311 |
| ***UBA7*** | 0.044309 |
| ***PCNT*** | 0.046014 |
| ***PPP1R21*** | 0.046443 |
| ***CIC*** | 0.047902 |
