# Supplementary material for: Integrative Analysis of Proteomics and DNA Methylation in Orbital Fibroblasts From Graves’ Ophthalmopathy
Source: Front Endocrinol (Lausanne). 2021 Feb 15;11:619989. doi: 10.3389/fendo.2020.619989 (PMC7919747; doi:10.3389/fendo.2020.619989)
Supplement: Supplementary file 7 [file Table_3.docx]

**Supplementary table 3** List of hypermethylated genes with the cut-off at more than 2-fold difference in the active GO orbital fibroblasts

| Gene | ID Illumina | Fold Difference |
| --- | --- | --- |
| WDR8 | cg26422465 | 6.921524187 |
| C16orf75 | cg27226511 | 3.894583048 |
| LZTS2 | cg25096861 | 3.885744689 |
| SPAG1 | cg07518837 | 3.507199303 |
| WDSUB1 | cg07508773 | 3.444116483 |
| NAAA | cg08223534 | 3.442807214 |
| FADS3 | cg22837486 | 3.408962486 |
| MYOM2 | cg21847720 | 3.27602843 |
| EGR1 | cg01290504 | 3.131284659 |
| PCBD1 | cg05601623 | 3.117146532 |
| KCNAB1 | cg01800345 | 3.062626463 |
| FGF18 | cg02125316 | 2.922885576 |
| MAB21L1 | cg16877681 | 2.882939862 |
| SPAG1 | cg13687825 | 2.798118042 |
| KCNJ10 | cg05768141 | 2.770041021 |
| TRIM2 | cg07318284 | 2.765862584 |
| SERTAD3 | cg14150973 | 2.717718168 |
| PFDN4 | cg13347296 | 2.710178468 |
| DGKQ | cg00741675 | 2.691331121 |
| SPAG1 | cg11067736 | 2.691037474 |
| RXFP3 | cg12238343 | 2.667350956 |
| NADSYN1 | cg00268518 | 2.66104792 |
| LIN52 | cg23171812 | 2.634137112 |
| FHIT | cg17573813 | 2.631986759 |
| TMEM48 | cg17002138 | 2.619754287 |
| ZNF234 | cg00843105 | 2.614245412 |
| TTLL7 | cg10977910 | 2.576460775 |
| HLA-H | cg26375284 | 2.574211271 |
| CBX7 | cg13306870 | 2.559225898 |
| ZNF667 | cg07967091 | 2.527151674 |
| MIR548F5 | cg12029639 | 2.504277927 |
| KIF26A | cg24706981 | 2.495814415 |
| MAB21L1 | cg03356172 | 2.492065032 |
| MAB21L1 | cg12178237 | 2.490181194 |
| TRIM2 | cg19717235 | 2.487530891 |
| PPM1A | cg05225909 | 2.48746827 |
| MIR548F5 | cg03014934 | 2.468999735 |
| CHSY1 | cg25114630 | 2.467851134 |
| ITIH3 | cg05393861 | 2.455488051 |
| MAB21L1 | cg10587183 | 2.446195777 |
| NIPAL2 | cg15264255 | 2.435913449 |
| MYOM2 | cg01295646 | 2.414759326 |
| HOXD1 | cg19180624 | 2.402777297 |
| MLH1 | cg03901257 | 2.384598342 |
| ATP5G1 | cg25450121 | 2.3813774 |
| C1orf52 | cg21245975 | 2.375670464 |
| C2CD4A | cg20639658 | 2.349710744 |
| ZNF234 | cg25395304 | 2.345914841 |
| GABRA4 | cg12307698 | 2.334188584 |
| OR2L13 | cg03748376 | 2.32830583 |
| DGKQ | cg19408572 | 2.327463927 |
| MIR548F5 | cg12573705 | 2.297696735 |
| GPR6 | cg05756933 | 2.296501682 |
| SLIT2 | cg19940312 | 2.283638225 |
| LBX2 | cg00573355 | 2.282060524 |
| BTBD11 | cg15048554 | 2.279662463 |
| MIR548F5 | cg13446906 | 2.27388391 |
| WDR37 | cg26445189 | 2.27064216 |
| MCM6 | cg07169764 | 2.264920948 |
| LOC401097 | cg12177207 | 2.257698944 |
| EIF2AK3 | cg26347887 | 2.244276711 |
| BCAP29 | cg07196124 | 2.242140478 |
| MIR548F5 | cg22855860 | 2.231659764 |
| DTNBP1 | cg14385438 | 2.225197414 |
| UNC13A | cg22989649 | 2.22409399 |
| FZD1 | cg25336926 | 2.216488669 |
| HDGFRP3 | cg04537729 | 2.213160846 |
| MYOM2 | cg11424828 | 2.212068156 |
| ZFP64 | cg01529637 | 2.211933864 |
| PAX6 | cg14002345 | 2.211862241 |
| TTC12 | cg12177743 | 2.207466273 |
| NKX3-1 | cg18502021 | 2.206682742 |
| PRDM7 | cg05270750 | 2.206033262 |
| NBLA00301 | cg15195321 | 2.20596421 |
| DKK1 | cg08812555 | 2.198207258 |
| DAXX | cg05431670 | 2.197888937 |
| SKP2 | cg19135982 | 2.196728374 |
| GNG7 | cg08994082 | 2.186547316 |
| ADAP1 | cg19665696 | 2.184918234 |
| LHX2 | cg14093715 | 2.184776116 |
| FAM110A | cg11979743 | 2.180324577 |
| FLNB | cg06793267 | 2.173961601 |
| LOC100128811 | cg25124276 | 2.165143768 |
| GPR6 | cg15381304 | 2.163764854 |
| TMEM87A | cg19391239 | 2.160638809 |
| ANKRD11 | cg27183791 | 2.160234314 |
| RPH3AL | cg23246911 | 2.156739993 |
| FAM162B | cg13631916 | 2.156720212 |
| TAGLN3 | cg19778003 | 2.156278594 |
| CPT1A | cg03855388 | 2.154290122 |
| MAB21L1 | cg00505001 | 2.152722394 |
| CTSZ | cg06385087 | 2.152294277 |
| RPH3AL | cg10440639 | 2.138455359 |
| C15orf42 | cg24528923 | 2.137085479 |
| LOC440356 | cg03397234 | 2.133232569 |
| FAM134A | cg10090279 | 2.132273121 |
| OBFC1 | cg11954230 | 2.12933873 |
| SLC45A3 | cg11455040 | 2.128743757 |
| TMEM87B | cg19917083 | 2.12347447 |
| DBR1 | cg12823953 | 2.123310552 |
| CPLX2 | cg13564889 | 2.119091558 |
| MIR196B | cg05250768 | 2.117170584 |
| CAMK1D | cg12273284 | 2.106540203 |
| FOXJ1 | cg05162533 | 2.103659451 |
| IER2 | cg14567593 | 2.10350386 |
| GDF6 | cg17999376 | 2.102409875 |
| NTF3 | cg00252282 | 2.101515721 |
| FBXO16 | cg18239973 | 2.09403245 |
| NBEA | cg00729885 | 2.089679306 |
| RPH3AL | cg11940040 | 2.083609609 |
| MIR548F5 | cg21884062 | 2.081909121 |
| AHNAK2 | cg01513078 | 2.081722403 |
| MAK16 | cg01581037 | 2.078323241 |
| RNF207 | cg16094412 | 2.074970358 |
| PAQR5 | cg13117105 | 2.074093749 |
| NXN | cg13417862 | 2.066925564 |
| GNAO1 | cg09432792 | 2.063068718 |
| KIAA1143 | cg24858591 | 2.061934647 |
| OR2L13 | cg04028570 | 2.059263 |
| OR2L13 | cg20507276 | 2.054976185 |
| CAMTA1 | cg10989261 | 2.052787598 |
| DMTF1 | cg05757385 | 2.041775676 |
| MAB21L1 | cg05093686 | 2.039949774 |
| C11orf20 | cg09675196 | 2.038711989 |
| SDHAP3 | cg08422420 | 2.036950987 |
| PAQR5 | cg22397562 | 2.036066567 |
| TRIM2 | cg22127901 | 2.033958701 |
| C17orf51 | cg18532727 | 2.029233739 |
| CHERP | cg02010894 | 2.028588014 |
| GRID2 | cg06855422 | 2.028200736 |
| RNF141 | cg05367858 | 2.026339503 |
| RMST | cg02716635 | 2.018661806 |
| PGCP | cg09533869 | 2.0173521 |
| NAV2 | cg02367892 | 2.014791536 |
| NIPAL2 | cg01949798 | 2.014715806 |
| CD248 | cg16182743 | 2.011908585 |
| MATN2 | cg08977887 | 2.009968231 |
| BARHL1 | cg14651249 | 2.007790438 |
| TTC12 | cg05127217 | 2.004017882 |
| HTATIP2 | cg24426391 | 2.003486295 |
| HTATIP2 | cg02900213 | 2.001568353 |
| SGOL2 | cg05409597 | 2.000174832 |
