# Supplementary material for: Integrative Analysis of Proteomics and DNA Methylation in Orbital Fibroblasts From Graves’ Ophthalmopathy
Source: Front Endocrinol (Lausanne). 2021 Feb 15;11:619989. doi: 10.3389/fendo.2020.619989 (PMC7919747; doi:10.3389/fendo.2020.619989)
Supplement: Supplementary file 8 [file Table_4.docx]

**Supplementary table 4** List of hypomethylated genes with the cut-off at more than 2-fold difference in the active GO orbital fibroblasts

| Gene | ID Illumina | Fold Difference |
| --- | --- | --- |
| RNF168 | cg01550828 | 5.079979155 |
| ERN2 | cg16364709 | 4.53742047 |
| BZW1 | cg03193689 | 3.767354176 |
| KCTD18 | cg27216630 | 3.733055246 |
| MYADML | cg04131969 | 3.371426016 |
| AP1M1 | cg26701807 | 3.362624016 |
| CMTM8 | cg19726630 | 3.202355265 |
| PRKCB | cg08406370 | 3.139585427 |
| PSMD2 | cg04520948 | 3.135435756 |
| TMEM120A | cg25286967 | 3.121801449 |
| EFHD2 | cg24070543 | 3.006689314 |
| LIPT1 | cg15544633 | 2.975798342 |
| HTR1A | cg13666507 | 2.944339743 |
| SCUBE2 | cg12150991 | 2.925586751 |
| FLJ16779 | cg06763829 | 2.860893312 |
| TM9SF1 | cg23418075 | 2.775724861 |
| ALS2CR4 | cg16678169 | 2.687301434 |
| CAPRIN2 | cg26378403 | 2.681358517 |
| PHLDB1 | cg15392109 | 2.665767958 |
| SCMH1 | cg14498674 | 2.659809761 |
| AMACR | cg20381404 | 2.657007422 |
| RPN1 | cg20294181 | 2.540821753 |
| TMEM176B | cg22962698 | 2.522387685 |
| LCLAT1 | cg17749961 | 2.50373035 |
| MXRA7 | cg03810198 | 2.481990599 |
| NFIC | cg08380311 | 2.45585253 |
| EFEMP1 | cg25268863 | 2.421404595 |
| PHLDB2 | cg20131219 | 2.385324357 |
| RAP1GDS1 | cg14454094 | 2.357794779 |
| SEC24D | cg21605333 | 2.353754819 |
| AIRE | cg27251412 | 2.351032546 |
| FZD5 | cg14011663 | 2.34125105 |
| AIRE | cg09510531 | 2.336573319 |
| TNIK | cg22901347 | 2.320818162 |
| C5orf33 | cg09819502 | 2.296625589 |
| HLA-A | cg11722179 | 2.265727573 |
| IAH1 | cg05986044 | 2.261393885 |
| CR1L | cg21106486 | 2.238161582 |
| SKAP1 | cg24636368 | 2.237148513 |
| HRNBP3 | cg04487202 | 2.221680413 |
| HLA-A | cg21591486 | 2.221369337 |
| SIM2 | cg15316660 | 2.185556161 |
| WDR55 | cg25613667 | 2.180410978 |
| ROR1 | cg17171259 | 2.171555302 |
| FRG1B | cg14815891 | 2.165138389 |
| TOX2 | cg26365090 | 2.163717508 |
| ACAD9 | cg22925115 | 2.155190752 |
| WNK4 | cg05878104 | 2.148109276 |
| REPS1 | cg07184423 | 2.139482731 |
| DPP9 | cg00371891 | 2.138500528 |
| PSORS1C1 | cg19456997 | 2.136183018 |
| TRIM61 | cg08992305 | 2.13444258 |
| GPR98 | cg02251859 | 2.122335367 |
| LRRC24 | cg08872590 | 2.105182026 |
| KCNJ9 | cg15580417 | 2.099856266 |
| ZNF562 | cg21610904 | 2.096149294 |
| FAM19A5 | cg24094706 | 2.091669602 |
| OSGIN1 | cg07452659 | 2.088976966 |
| HS3ST2 | cg00885682 | 2.075058283 |
| GAL3ST3 | cg26763523 | 2.071077135 |
| SIN3A | ch.15.1221715R | 2.050917655 |
| COLEC11 | cg10724632 | 2.034703298 |
| PRKCDBP | cg20938665 | 2.032961325 |
| SIM2 | cg23286646 | 2.028335275 |
| CLIC6 | cg16628066 | 2.003347837 |
| PPP1R7 | cg16751493 | 2.002133866 |
| PACSIN2 | cg02940070 | 1.996936188 |
| ERICH1 | cg12641240 | 1.992493672 |
| CDH15 | cg27202913 | 1.981773733 |
| ENTPD3 | cg10755058 | 1.967849383 |
| NRG1 | cg25050565 | 1.967078072 |
| CDC42BPB | ch.14.1795106F | 1.96582915 |
